# Gaps in Healthcare Seeking for Childhood Diarrhea Among Peruvian Children Under Five Years of Age: Socioeconomic and Clinical Patterns in a National Survey

**DOI:** 10.3390/children13101300

**Published:** 2026-09-25

**Authors:** Víctor Juan Vera-Ponce, Jhosmer Ballena-Caicedo, Namibia Jherly Cervera Vasquez, Marcos García-Rodríguez, Jorge Alejandro Yumpo Ynga, Fiorella E. Zuzunaga-Montoya

**Affiliations:** Facultad de Medicina (FAMED), Universidad Nacional Toribio Rodríguez de Mendoza de Amazonas (UNTRM), Chachapoyas 01001, Peru; 7330178022@untrm.edu.pe (J.B.-C.); 7679509541@untrm.edu.pe (N.J.C.V.); marcos.garcia@untrm.edu.pe (M.G.-R.); 7215802831@untrm.edu.pe (J.A.Y.Y.); fiorella.zuzunaga@untrm.edu.pe (F.E.Z.-M.)

**Keywords:** childhood diarrhea, child, care seeking, health services, oral rehydration salts, zinc, Peru

## Abstract

**Highlights:**

**What are the main findings?**

- In Peru during 2019–2024, 43.5% of recent childhood diarrhea episodes involved no external advice, care, or treatment, and only 33.1% reached a restrictive formal source.
- Therapeutic coverage was low: 34.3% received oral rehydration salts, 1.0% zinc, and 0.5% both; non-use was more frequent among older children and those in the poorest households, but less frequent when blood in stool or fever was reported.

**What are the implications of the main findings?**

- Care pathways should combine caregiver recognition of warning features with reliable access to oral rehydration salts and zinc, including through pharmacies and other non-formal sources.
- Monitoring should distinguish any external contact, formal care, and evidence-based treatment, because contact with a service does not necessarily ensure appropriate diarrhea management.

**Abstract:**

Background: Childhood diarrhea remains a preventable public health problem, but gaps persist in contact with external care and receipt of effective treatment. Methods: We estimated the prevalence of non-use of external care for recent diarrhea among Peruvian children aged 0–59 months during 2019–2024 and examined associated clinical, maternal, socioeconomic, and territorial factors. We analyzed repeated cross-sectional ENDES microdata using survey-weighted modified Poisson regression with Taylor-linearized variance, year-specific primary sampling units, and strata. Results: Among 11,942 child-level survey observations with classifiable care-seeking information, non-use of external care was 43.5% (95% CI: 42.2–44.8); restrictive formal care seeking was 33.1%, and 23.4% (95% CI: 22.3–24.5) used only non-formal external sources. Among observations with valid treatment-specific information, 34.3% received oral rehydration salts, 1.0% zinc, and 0.5% both. Blood in stool was associated with lower non-use (aPR = 0.52; 95% CI: 0.42–0.63), whereas age 48–59 months was associated with higher non-use than age 0–11 months (aPR = 1.20; 95% CI: 1.08–1.33). The poorest quintile had modestly higher non-use than the richest quintile (aPR = 1.20; 95% CI: 1.03–1.41). Non-use was not associated with diarrhea still active at interview in the prespecified secondary model (PR = 0.99; 95% CI: 0.87–1.12). Conclusions: More than two in five recent episodes had no external care contact, and therapeutic coverage remained limited, particularly for zinc.

## 1. Introduction

Diarrheal diseases remain a preventable and treatable cause of childhood morbidity and mortality. Globally, nearly 1.7 billion episodes of childhood diarrhea are estimated each year, and diarrhea causes approximately 444,000 deaths annually among children under five years of age; it also accounts for about 9% of deaths in this age group. The relevance of the problem is not limited to mortality: repeated episodes are associated with dehydration, nutritional deterioration, greater vulnerability to new infections, and additional pressure on households and health services [1,2].

Timely management of childhood diarrhea is a high-value health intervention because it combines prevention measures, early recognition of severity, and effective treatment. Oral rehydration salts and zinc are low-cost interventions recommended for the treatment of acute childhood diarrhea; they reduce episode duration and severity, decrease the short-term risk of recurrence, and require timely access to treatment or appropriate health guidance [1,3]. However, treatment coverage remains insufficient: UNICEF reports that the use of oral rehydration salts and zinc remains below expected levels, with persistent gaps by poverty, rurality, and service availability [2].

In Latin America and the Caribbean, diarrheal mortality has declined substantially in recent decades, but fewer deaths do not mean that the epidemiological problem has been resolved. Regional analyses show sustained declines in mortality between 2000 and 2019, although with heterogeneity across countries and persistent burden among vulnerable populations [4]. In addition, evidence from poor and Indigenous communities in Mesoamerica has documented recent diarrhea prevalence, low adherence to evidence-based interventions, limited zinc use, and nontrivial antibiotic use, suggesting that the current challenge is not only to prevent episodes but also to ensure timely and appropriate management when they occur [5].

In Peru, childhood diarrhea has mainly been studied as an occurrence event, with emphasis on its socioeconomic, environmental, and territorial determinants. A recent national analysis reported a diarrhea prevalence of 10.0% among children under five years of age in 2021 and showed a pro-poor distribution, with greater concentration in the Highlands and the Jungle [6]. This evidence is useful for identifying where diarrhea occurs; however, it leaves less clear what happens after the episode: who does not seek external care, which groups reach or fail to reach the system, and to what extent children receive recommended therapeutic interventions. This gap is methodologically relevant because diarrhea occurrence, care seeking, and quality of management belong to distinct links in the health response.

Importantly, non-use of external care should not be interpreted as implying that every diarrheal episode requires facility-based medical consultation. Many uncomplicated episodes can be managed at home when caregivers receive adequate guidance and have access to recommended treatment. The epidemiological concern addressed here is the absence of any external source of advice, care, or treatment, because this may limit timely recognition of warning features, access to oral rehydration salts and zinc, and referral when symptoms suggest greater clinical need [1,3].

This study provides updated national evidence by shifting the focus from diarrhea prevalence to the post-episode care pathway. The primary objective was to estimate the prevalence of non-use of external care for recent diarrhea among boys and girls under five years of age in Peru during 2019–2024 and to evaluate its association with markers of clinical need, child characteristics, maternal factors, and enabling access conditions. Secondary objectives were to estimate the use of formal and non-formal external sources, characterize therapeutic management—including oral rehydration salts, zinc, antibiotics, and home remedies—and explore the cross-sectional association between non-use of care and diarrhea still active at interview.

## 2. Materials and Methods

### 2.1. Study Design

We conducted an analytical cross-sectional observational study using secondary microdata from the Peruvian Demographic and Family Health Survey (ENDES). The analysis integrated six consecutive annual rounds, corresponding to 2019–2024, and was restricted to boys and girls under 5 years of age with available information on recent diarrhea. The manuscript was prepared following the STROBE recommendations for observational studies, with emphasis on transparent reporting of the data source, sample selection, operational definitions, and management of the complex sampling design [7].

The analytical objective was to estimate the frequency of non-use of external care for recent episodes of childhood diarrhea and to evaluate its association with markers of clinical need, child characteristics, maternal characteristics, and enabling access factors. Given the cross-sectional design, associations were interpreted as measures of disparity and not as causal effects.

### 2.2. Data Source

ENDES is a population-based survey conducted continuously by the Peruvian National Institute of Statistics and Informatics (INEI). Its purpose is to produce updated information on demographic dynamics, maternal and child health, communicable and noncommunicable diseases, and access to diagnostic and treatment services [8]. The microdata, questionnaires, and methodological documents are publicly available through the INEI institutional portal.

The survey uses a complex, probabilistic, stratified, multistage sampling design, with selection of clusters and households within study domains. Data collection is performed through direct interviews by trained personnel, using standardized questionnaires administered in private households. For this study, we used variables from the child and household modules related to diarrhea in the two weeks preceding the interview, sources of care or treatment, treatments received, sociodemographic characteristics, and sampling design variables.

### 2.3. Population and Sample

The unit of analysis was a child-level survey observation corresponding to a boy or girl aged 0–59 months with caregiver-reported diarrhea in the two weeks preceding the interview. ENDES is a repeated cross-sectional household survey and not a patient registry; therefore, the term “children” refers to sampled child-level observations. Public-use files are anonymized, and child-level observations cannot be linked across annual rounds. Repeated inclusion of the same individual across years therefore cannot be assessed empirically, although ENDES is designed to produce annual population estimates rather than longitudinal follow-up.

For the main analysis, we included child-level observations with recent diarrhea and classifiable information on external care seeking. The secondary outcome sample was restricted to observations with a valid H44C response indicating whether diarrhea was still present at interview. Complete-case analysis was used for multivariable models. Records excluded because of missing or non-classifiable information were retained during data preparation to audit selection and missingness. Variable-specific missingness and treatment denominators are detailed in Supplementary Table S4.

### 2.4. Variables

The main outcome was non-use of external care for childhood diarrhea. Variables H12A–H12X and H12Z record source-specific caregiver reports of advice, care, or treatment for the diarrheal episode, whereas H12Y indicates no advice, care, or treatment. External care seeking was classified when at least one H12A–H12X/H12Z source was affirmative. Non-use was classified when no external source was affirmative and H12Y indicated no care or treatment. A source-specific affirmative response was prespecified to take precedence if a conflict occurred; however, the audit identified no contradictory records. The 238 unclassifiable observations had no affirmative external source and missing or non-informative H12Y data. Restrictive formal care excluded pharmacy, drugstore, direct-purchase, non-medical, and traditional sources; non-formal-only care comprised external care seeking without a restrictive formal source. Full source mappings are provided in Supplementary Table S2. Alternative care-seeking indicators and care-cascade stages are detailed in Supplementary Table S5.

Secondary outcomes were derived from caregiver-reported ENDES variables referring to the same recent diarrheal episode. Oral rehydration salts were defined from H13/H13B. The primary zinc definition used the populated zinc-specific variables S473DA and S473DB; H15E was examined only as a sensitivity because its label in the integrated file conflicted with the official dictionary. Antibiotic receipt was defined from H15/H15B according to the official INEI dictionary; H15A was retained separately as diarrhea syrup. Time to care was obtained from H44B, which records days from diarrhea onset to advice or treatment. Diarrhea still active was defined from H44C, whose questionnaire wording asks whether the child “still has diarrhea” at interview. H46B was not used because it records the interval before seeking advice or treatment for fever, not continuity of diarrhea.

Markers of clinical need were blood in stool and co-reported fever during the reference period. Fever was treated as a temporal co-report and not as clinical confirmation of fever attributable to the diarrheal episode. For descriptive stratification, survey-defined warning features were defined as blood in stool and/or co-reported fever. This indicator is not a complete clinical danger-sign assessment because ENDES does not measure dehydration, lethargy, inability to drink, persistent vomiting, or clinician-assessed severity.

Adjustment covariates were selected a priori from a conceptual framework of clinical need, predisposing factors, and enabling access factors: child age and sex, maternal age and education, parity, wealth quintile, area of residence, natural region, health insurance, survey year, and rainy season. Treatment variables were not included in the main care-seeking model because they may occur after external contact and act as mediators or consequences. Stunting and wasting were treated as background vulnerability factors, not episode-level warning features, and were evaluated in sensitivity models among observations with valid anthropometry.

### 2.5. Statistical Analysis

All analyses accounted for the ENDES complex sampling structure. Annual sampling weights were divided by six only to normalize the scale of the pooled 2019–2024 file; weighted totals were not interpreted as population counts. Primary sampling units and strata were made unique within a survey year. Estimation used StataNow/SE 19.5 survey commands with Taylor linearization, sampling weights, strata, and primary sampling units; singleton strata were centered. Finite population corrections were not applied because harmonized sampling fractions were unavailable. Subgroup estimates were obtained by domain analysis so that the full design contributed to variance estimation.

The sample was summarized with unweighted frequencies and survey-weighted percentages. Prevalences and design-based 95% confidence intervals were estimated for the main and secondary outcomes. Table 1 reports weighted column percentages, whereas category-specific row prevalences of non-use are provided in Supplementary Table S7. Two-sided *p*-values are reported in the regression tables, but interpretation prioritized effect size, direction, and precision rather than dichotomous significance thresholds.

For the main outcome, prevalence ratios were estimated using modified Poisson regression with a log link and survey-design-based covariance. This approach was selected because non-use of external care was frequent and prevalence ratios are more interpretable than odds ratios in this setting [9]. The main model included blood in stool, co-reported fever, child age and sex, maternal age and education, parity, wealth quintile, area, natural region, health insurance, survey year, and rainy season.

Adjusted marginal prevalences and adjusted prevalence differences were obtained by marginal standardization from the fitted modified Poisson model. Confidence intervals were calculated with the delta method using the full design-based covariance matrix of the regression coefficients, thereby propagating sampling weights, primary sampling units, and strata through the standardization step. A formal ordinal wealth-trend model and a warning-feature-by-wealth interaction model were fitted as supplementary analyses.

The secondary outcome of diarrhea still active was evaluated with sequential modified Poisson models: M0 included non-use of external care only; M1 additionally adjusted for blood in stool; M2 additionally adjusted for co-reported fever; M3 adjusted for both blood in stool and co-reported fever and was the prespecified primary secondary outcome model; and M4 added child, maternal, socioeconomic, geographic, year, and season covariates as a sensitivity analysis. These models were interpreted strictly as cross-sectional associations and not as effects of care seeking on episode duration.

Sensitivity and verification analyses included additional adjustment for stunting or wasting, alternative zinc and antibiotic source-variable definitions, year-specific ORS/zinc estimates, the H12 contradiction audit, H44B completeness, H11-by-H44C verification, non-formal-only source composition, wealth-trend and interaction tests, and survey-design diagnostics.

The primary prevalence calculation retained 17,978 primary sampling units in 1311 strata; 8287 primary sampling units contained observations in the recent diarrhea analytic domain. The design effect for the main prevalence estimate was 2.06. Detailed diagnostics are reported in Supplementary Table S7.

Primary estimation was performed in StataNow/SE 19.5 (StataCorp LLC, College Station, TX, USA). Python 3.12.0 (Python Software Foundation, Beaverton, OR, USA) was used only for output verification, figure rendering, and document generation, with pandas 3.0.3, NumPy 2.4.6, matplotlib 3.11.0, and python-docx 1.2.0; patsy 1.0.2 and statsmodels 0.14.6 were not used for final survey estimation. Full software details are provided in Supplementary Table S7.

### 2.6. Ethical Considerations

The study used exclusively anonymized, publicly available secondary microdata from the INEI portal. We had no access to names, identity documents, individual addresses, or other identifiers. Formal ethics committee review was not sought because the analysis involved no participant contact, no primary data collection, and no identifiable private information; no formal institutional review board waiver was issued. The analysis followed principles applicable to secondary data use, including preservation of confidentiality, respect for permitted data use, and avoidance of reidentification [10].

Primary ENDES data collection was performed by INEI under its institutional procedures for consent, confidentiality, and quality control [8]. The present analysis was limited to the statistical reuse of public data to answer a distinct epidemiological question, without modifying the original relationship between interviewer and participant.

## 3. Results

### 3.1. Sample Selection

Across the six annual ENDES rounds from 2019 to 2024, 100,119 children aged 0–59 months were identified. Of these, 87,939 had no recent diarrhea; 12,180 child-level survey observations were therefore eligible because diarrhea had been reported in the preceding two weeks. The original annual REC43 files contained no observations coded H11 = 1 (diarrhea in the last 24 h); all affirmative recent diarrhea records were coded H11 = 2. After exclusion of 238 observations with unclassifiable care-seeking status, the main analytical sample comprised 11,942 observations. The main adjusted model included 11,493 complete cases, and the primary secondary outcome model included 11,921. The selection process is shown in Supplementary Figure S1.

### 3.2. Characteristics of the Study Population

The weighted prevalence of non-use of external care was 43.5% (95% CI: 42.2–44.8), whereas 56.5% reported at least one external source. Category-specific non-use was lower with blood in stool than without it (22.1% vs. 44.8%), lower with co-reported fever than without it (37.4% vs. 48.0%), and lower with survey-defined warning features than without them (36.6% vs. 49.0%). Non-use was 45.1% in the poorest quintile and 39.6% in the richest quintile. Table 1 reports weighted column distributions; the corresponding row percentages and design-based intervals are provided in Supplementary Table S7.

### 3.3. Prevalence of Care Seeking and Therapeutic Management

Restrictive formal care seeking was 33.1%. In addition, 23.4% of children (95% CI: 22.3–24.5) used only non-formal external sources without reaching a restrictive formal source; this represented 41.4% (95% CI: 39.7–43.1) of those who sought any external care. The non-formal-only group consisted mainly of pharmacy or botica sources (76.4%), followed by friends or relatives (22.8%). Among observations with valid treatment-specific information, 34.3% received oral rehydration salts, 1.0% zinc, 0.5% both, 17.7% antibiotics, and 22.3% home remedies; 18.6% used a pharmacy or direct-purchase source. All 6690 external care seekers had valid H44B information, and 69.5% (95% CI: 67.9–71.0) sought care on the same day or the next day. Detailed denominators and intervals are provided in Supplementary Table S3.

### 3.4. Factors Associated with Non-Use of External Care

Figure 1 presents the conceptual framework. In the main multivariable model, blood in stool was associated with lower non-use of external care (aPR = 0.52; 95% CI: 0.42–0.63), as was co-reported fever (aPR = 0.80; 95% CI: 0.75–0.85). Compared with children aged 0–11 months, those aged 48–59 months had higher non-use (aPR = 1.20; 95% CI: 1.08–1.33). The poorest quintile had modestly higher non-use than the richest quintile (aPR = 1.20; 95% CI: 1.03–1.41; adjusted difference 7.8 percentage points, 95% CI: 1.5–14.1). Rural residence and lack of insurance did not show clear independent associations. Complete crude and adjusted estimates, *p*-values, marginal prevalences, and absolute differences are shown in Table 2.

### 3.5. Non-Use of External Care by Survey-Defined Warning Features and Wealth Quintile

Figure 2 shows the weighted prevalence of non-use of external care by survey-defined warning features and wealth quintile. Among children without warning features, estimates were non-monotonic, ranging from 46.1% in the richest quintile to 51.0% in the middle quintile. Among children with warning features, estimates were lower overall and ranged from 30.3% in the richest quintile to 40.2% in the poorest quintile. An ordinal wealth score showed a small association per one-level shift toward greater poverty (aPR = 1.04; 95% CI: 1.00–1.07; p-trend = 0.041), but the category-specific estimates were not strictly monotonic and only the poorest-versus-richest adjusted contrast clearly excluded the null. The global warning-feature-by-wealth interaction was not conclusive (*p* = 0.186). Figure 2 is therefore interpreted descriptively; all ten estimates and design-based 95% confidence intervals are reported in Supplementary Table S7.

### 3.6. Diarrhea Still Active at the Time of the Interview

The weighted prevalence of diarrhea still active at interview was 14.6% (95% CI: 13.7–15.6) among 11,933 observations with valid H44C information. H44C asks whether the child still has diarrhea; it does not measure total episode duration. Sequential estimates for non-use of external care remained close to the null: M0 PR = 0.95 (95% CI: 0.84–1.08), M1 PR = 0.96 (0.85–1.09), M2 PR = 0.99 (0.87–1.12), and the prespecified M3 model PR = 0.99 (0.87–1.12). The expanded M4 sensitivity model yielded PR = 1.00 (0.88–1.13). Thus, no statistically supported cross-sectional association was identified. This analysis remains secondary and non-causal.The sequential model estimates are summarized in Table 3.

**Table 3:** Sequential survey-design-adjusted associations between non-use of external care and diarrhea still active at interview.

Model	Adjustment Set	n	PR (95% CI)	*p*-Value
M0	Non-use of external care only	11,933	0.95 (0.84–1.08)	0.464
M1	M0 plus blood in stool	11,924	0.96 (0.85–1.09)	0.509
M2	M0 plus co-reported fever	11,930	0.99 (0.87–1.12)	0.826
M3 (primary)	M0 plus blood in stool and co-reported fever	11,921	0.99 (0.87–1.12)	0.848
M4 (sensitivity)	M3 plus child, maternal, socioeconomic, geographic, year, and season covariates	11,485	1.00 (0.88–1.13)	0.995

PR: prevalence ratio. M3, adjusted for blood in stool and co-reported fever, was the prespecified primary secondary outcome model. M4 was an expanded sensitivity model. H44C indicates diarrhea still present at interview and should not be interpreted as total duration or as a causal effect of care seeking.

### 3.7. Sensitivity and Verification Analyses

Of the 11,942 observations in the main sample, 11,493 had complete information for the adjusted model. Additional adjustment for stunting or wasting did not materially change the estimates for blood in stool, co-reported fever, or wealth; stunting itself was not associated with non-use (aPR = 0.99; 95% CI: 0.91–1.07), and wasting had an imprecise association (aPR = 0.71; 95% CI: 0.46–1.10). The H12 audit identified no internal contradictions, H44B was complete for all 6690 care seekers, and the original-file H11 audit confirmed the absence of H11 = 1 records across all six rounds. Year-specific treatment estimates, source-variable sensitivities, design diagnostics, and other verification analyses are reported in Supplementary Tables S3, S6 and S7.

## 4. Discussion

### 4.1. Main Findings

In this national analysis of ENDES 2019–2024, more than two in five child-level observations with recent diarrhea had no external care seeking or advice, approximately one-third reached a restrictive formal source, and nearly one-quarter used only non-formal external sources. Blood in stool and co-reported fever were associated with lower non-use, while age 48–59 months and residence in the poorest households were associated with higher non-use. Wealth-related differences were modest and the descriptive quintile pattern was non-monotonic; the warning-feature-by-wealth interaction was not statistically conclusive. Therapeutic coverage was limited: approximately one-third received oral rehydration salts, 1.0% received zinc, and 0.5% received both. Non-use of external care was not associated with diarrhea still active in the prespecified secondary model, which should not be interpreted causally.

### 4.2. Comparison with Previous Studies

Our results are consistent with international evidence that care seeking for childhood diarrhea remains incomplete even where low-cost interventions are available. A multinational analysis of 35 sub-Saharan African countries estimated pooled care seeking at 58.7%, implying that more than 40% received no external advice or treatment; this magnitude is broadly comparable with Peru, although it does not imply epidemiological equivalence across regions [11]. Studies in poor communities of Mesoamerica have likewise documented simultaneous gaps in recent diarrhea, care seeking, and evidence-based treatment [5].

The inverse association between survey-defined warning features and non-use of external care is consistent with evidence that caregivers seek help more often for visible or concerning symptoms. Blood in stool and other severity markers have been associated with higher treatment or care seeking in multinational analyses [12,13]. Nevertheless, a substantial proportion of children with warning features still had no external contact. The descriptive poorest-versus-richest contrast was larger in this stratum, but the global interaction test was not conclusive; therefore, the finding is presented as a descriptive pattern rather than effect modification.

The wealth-related pattern also requires restraint. The adjusted model showed a modest difference mainly between the poorest and richest households, and the ordinal trend was small. Category-specific estimates were not a simple graded dose–response. This extends Peruvian evidence of pro-poor inequality in diarrhea occurrence [6] to the care pathway without implying that every successive wealth quintile had a uniformly lower probability of non-use. Rural residence and insurance coverage were not strong independent predictors after adjustment, which may reflect the inability of coarse survey categories to capture effective distance, indirect costs, opening hours, supply availability, trust, and caregiver decision-making.

Therapeutic coverage should be interpreted separately from care seeking. ORS receipt was consistent with the historical increase in Peru from 3.6% in 1986 to 32% in 2015 [14], but zinc receipt and joint ORS-plus-zinc use remained extremely low. Zinc-specific variables S473DA/S473DB were populated in all six rounds, and year-specific estimates remained between 0.4% and 1.2%, reducing concern that the pooled 1.0% estimate was driven by a single round. Historical DHS estimates for Peru were also low—1.5% in 2009, 0.4% in 2011, and 0.9% in 2012—although they are not a contemporary benchmark [15]. The estimate represents caregiver-reported zinc receipt captured by ENDES, not an audit of prescription, dispensing, dosage, adherence, or household stock. The contrast with 53.3% ORS–zinc co-utilization reported in East Africa underscores contextual and measurement differences rather than direct comparability [16].

### 4.3. Public Health Implications

From a public health perspective, the findings support monitoring a care cascade rather than formal consultation alone: episode recognition, identification of warning features, timely external advice, contact with an appropriate source, availability of ORS and zinc, continued feeding, and referral when clinically required. The 23.4% who used only non-formal sources are particularly actionable because pharmacies, boticas, relatives, and other community channels may be the first—and sometimes only—point of contact. Policies should therefore combine caregiver education with standardized counseling, referral pathways, and reliable supplies across facilities and community-facing channels.

Formal insurance should not be equated with effective access. The absence of a strong independent association between insurance and non-use suggests that nominal coverage alone does not resolve rapid care-seeking decisions or treatment quality. Evidence from Countdown analyses indicates that contact with facilities or community providers may improve appropriate management, while also showing that contact does not guarantee optimal treatment [13]. Interventions should therefore address operating hours, distance, indirect costs, provider training, supply continuity, and coordination with pharmacies and community providers.

Antibiotic receipt was 17.7%. ENDES does not measure indication, pathogen etiology, prescription status, dosage, or appropriateness, so this estimate cannot be classified as rational or unnecessary use. Nevertheless, antibiotic exposure in childhood diarrhea warrants monitoring because avoidable antimicrobial use can create adverse effects, unnecessary costs, and antimicrobial-resistance pressure [17]. The Peruvian case illustrates a broader transition challenge: child survival has improved, but effective coverage and therapeutic quality remain incomplete.

### 4.4. Limitations

This study has limitations. First, the cross-sectional design prevents causal temporality and creates risks of reverse causality and confounding by indication, especially for diarrhea still active. Second, episodes, treatments, and care sources were caregiver-reported and are susceptible to recall and classification error. Third, fever was a temporal co-report rather than clinically attributed fever. Fourth, H44C identifies whether diarrhea was still present at interview but does not measure total duration, dehydration, etiology, or objective severity; the original annual files contained no H11 = 1 records, so a last-24 h subgroup could not be compared. Fifth, treatment indicators had variable-specific denominators, and the survey does not assess prescription quality, dose, adherence, supply availability, indirect costs, or reasons for non-use. Sixth, discrepancies between integrated file labels and official dictionaries required transparent source-variable sensitivity analyses for zinc and antibiotics. Finally, results depend on harmonization across rounds and complete-case assumptions, although full survey-design estimation and multiple audits supported the robustness of the main findings.

## 5. Conclusions

In conclusion, during 2019–2024, 43.5% of Peruvian child-level observations with recent diarrhea and classifiable care-seeking information had no external care seeking or advice. Survey-defined warning features were associated with lower non-use, while wealth-related differences were modest and non-monotonic; the poorest quintile had higher adjusted non-use than the richest, but the warning-feature-by-wealth interaction was not conclusive. A further 23.4% used only non-formal external sources. ORS coverage remained limited, zinc receipt was 1.0%, and joint ORS–zinc receipt was 0.5%. Non-use of external care was not associated with diarrhea still active in the secondary cross-sectional analysis.

Interventions should strengthen caregiver recognition of warning features and early home management with ORS, ensure reliable ORS and zinc availability in facilities and community channels, improve counseling and referral from pharmacies and other non-formal sources, and monitor antibiotic exposure and care cascade indicators. Prospective and implementation studies are needed to measure timeliness, reasons for non-use, treatment appropriateness, supply continuity, adherence, and clinical outcomes.

## Figures and Tables

**Figure 1:**
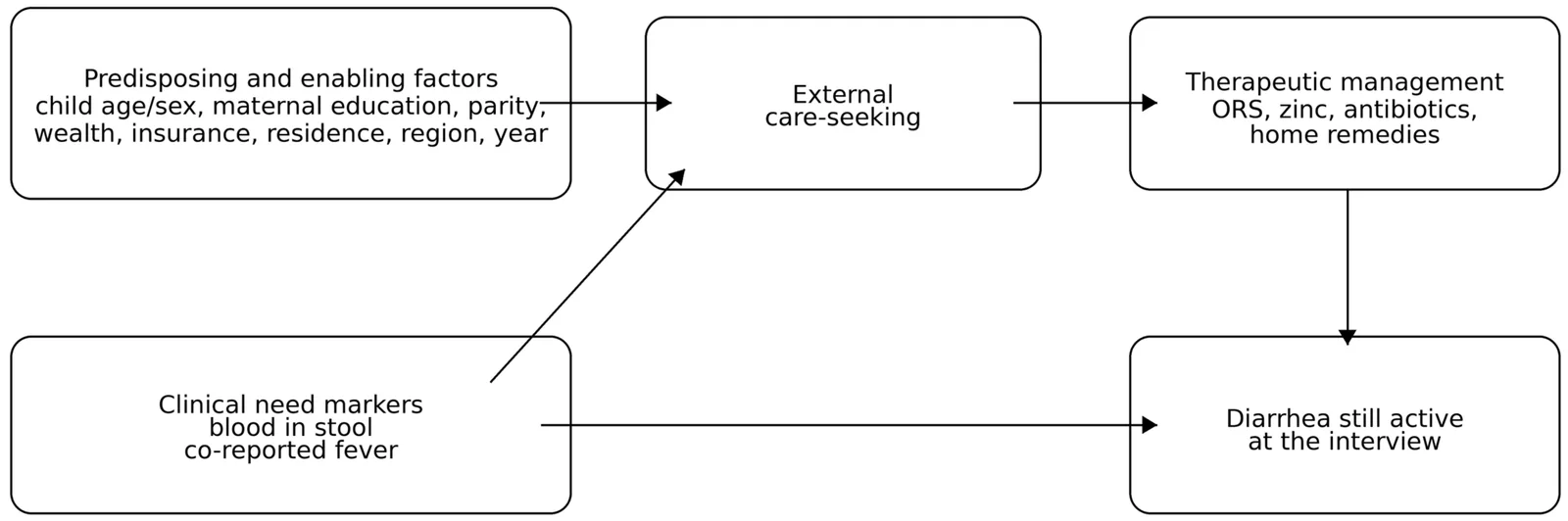
Conceptual framework for the analysis of non-use of external care, therapeutic management, and diarrhea still active. Arrows indicate conceptual relationships used to organize the analysis and should not be interpreted as tested mediation pathways.

**Figure 2:**
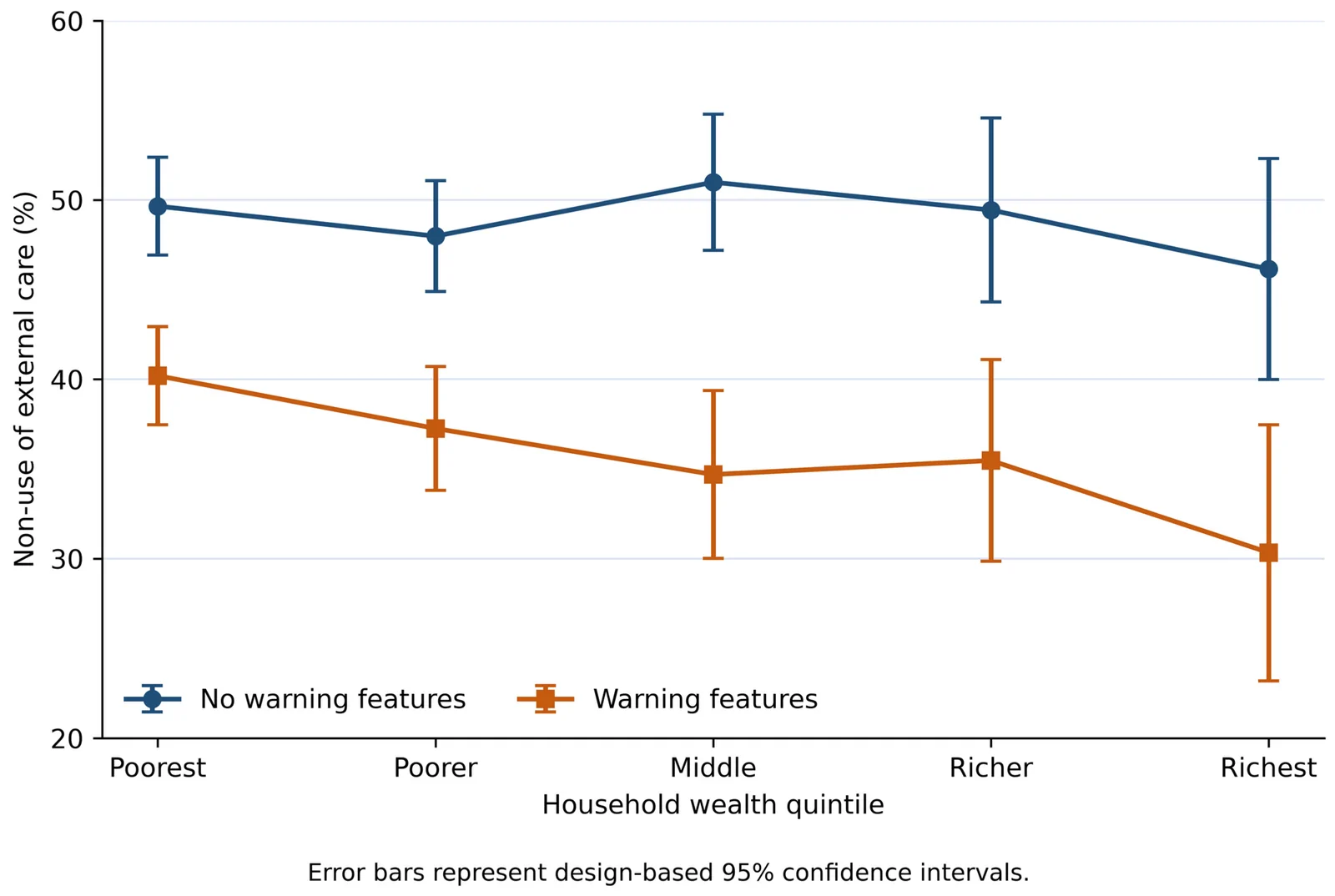
Weighted prevalence of non-use of external care by survey-defined warning features and wealth quintile. Error bars represent design-based 95% confidence intervals.

**Table 1:** Characteristics of children under 5 years of age with recent diarrhea by external care seeking, ENDES 2019–2024.

Variable/Category	Total	Sought External Care	Did Not Seek External Care
**Child age**			
0–11 months	2624 (22.0)	1467 (22.3)	1157 (21.6)
12–23 months	4005 (32.1)	2392 (34.1)	1613 (29.6)
24–35 months	2604 (22.4)	1464 (22.5)	1140 (22.2)
36–47 months	1597 (14.1)	823 (13.0)	774 (15.6)
48–59 months	1112 (9.4)	544 (8.1)	568 (11.0)
**Child sex**			
Male	6235 (52.3)	3524 (53.1)	2711 (51.3)
Female	5707 (47.7)	3166 (46.9)	2541 (48.7)
**Blood in stool**			
No	11,231 (94.4)	6151 (92.2)	5080 (97.1)
Yes	702 (5.6)	535 (7.8)	167 (2.9)
**Co-reported fever**			
No	6942 (58.2)	3573 (53.6)	3369 (64.1)
Yes	4997 (41.8)	3114 (46.4)	1883 (35.9)
**Survey-defined warning features**			
No	6627 (55.8)	3341 (50.4)	3286 (62.9)
Yes	5315 (44.2)	3349 (49.6)	1966 (37.1)
**Maternal age**			
15–19 years	2144 (17.0)	1172 (16.6)	972 (17.5)
20–34 years	7457 (62.1)	4219 (62.6)	3238 (61.4)
35–49 years	2264 (20.9)	1255 (20.8)	1009 (21.1)
**Maternal education**			
None	89 (0.6)	55 (0.7)	34 (0.5)
Primary	2068 (16.3)	1148 (16.1)	920 (16.7)
Secondary	6231 (51.6)	3426 (49.9)	2805 (53.9)
Higher	3477 (31.4)	2017 (33.3)	1460 (28.9)
**Parity**			
1 child	2993 (20.9)	1728 (21.2)	1265 (20.4)
2–3 children	6143 (57.5)	3413 (57.7)	2730 (57.2)
≥4 children	2444 (21.6)	1342 (21.1)	1102 (22.3)
**Wealth quintile**			
Poorest	3895 (25.8)	2145 (25.1)	1750 (26.8)
Poorer	3576 (27.4)	2031 (27.6)	1545 (27.2)
Middle	2246 (20.6)	1238 (20.4)	1008 (20.8)
Richer	1407 (15.1)	786 (15.1)	621 (15.1)
Richest	818 (11.0)	490 (11.8)	328 (10.0)
**Area of residence**			
Urban	8044 (75.7)	4541 (76.1)	3503 (75.0)
Rural	3898 (24.3)	2149 (23.9)	1749 (25.0)
**Natural region**			
Metropolitan Lima	1161 (25.2)	618 (24.0)	543 (26.8)
Coast	3006 (25.1)	1694 (26.1)	1312 (23.8)
Highlands	3873 (27.0)	2072 (26.6)	1801 (27.6)
Jungle	3902 (22.7)	2306 (23.4)	1596 (21.8)
**Health insurance**			
No	1191 (9.9)	654 (9.4)	537 (10.6)
Yes	10,751 (90.1)	6036 (90.6)	4715 (89.4)
**Survey year**			
2019	2206 (13.2)	1178 (12.6)	1028 (13.9)
2020	941 (6.6)	509 (6.2)	432 (7.2)
2021	1887 (15.6)	1071 (16.3)	816 (14.7)
2022	2334 (21.8)	1368 (22.6)	966 (20.8)
2023	2420 (22.9)	1405 (23.2)	1015 (22.6)
2024	2154 (19.9)	1159 (19.1)	995 (20.8)
**Rainy season**			
No	8153 (68.9)	4631 (70.0)	3522 (67.5)
Yes	3789 (31.1)	2059 (30.0)	1730 (32.5)

Cells show unweighted *n* and weighted percentage within each column. Denominators vary because of missing data: blood in stool, *n* = 11,933; co-reported fever, *n* = 11,939; maternal age and maternal education, *n* = 11,865; parity, *n* = 11,580. Survey-defined warning features were classifiable for all 11,942 observations. Category-specific row prevalences of non-use are reported in Supplementary Table S7.

**Table 2:** Factors associated with non-use of external care among children under 5 years of age with recent diarrhea, ENDES 2019–2024.

Variable/Category	Crude PR (95% CI; *p*)	Adjusted PR (95% CI; *p*)	Adjusted Prevalence, % (95% CI)	Adjusted Difference, pp (95% CI; *p*)
**Blood in stool**				
No	1.00 (ref.)	1.00 (ref.)	44.6 (43.3–45.9)	0.0 (ref.)
Yes	0.49 (0.40–0.60); *p* < 0.001	0.52 (0.42–0.63); *p* < 0.001	23.0 (18.5–27.5)	−21.6 (−26.3 to −17.0); *p* < 0.001
**Co-reported fever**				
No	1.00 (ref.)	1.00 (ref.)	47.4 (45.7–49.1)	0.0 (ref.)
Yes	0.78 (0.73–0.83); *p* < 0.001	0.80 (0.75–0.85); *p* < 0.001	37.7 (35.7–39.7)	−9.7 (−12.3 to −7.1); *p* < 0.001
**Child age**				
0–11 months	1.00 (ref.)	1.00 (ref.)	42.7 (40.0–45.4)	0.0 (ref.)
12–23 months	0.94 (0.86–1.02); *p* = 0.118	0.94 (0.87–1.02); *p* = 0.133	40.1 (38.0–42.2)	−2.6 (−5.9 to 0.8); *p* = 0.136
24–35 months	1.01 (0.93–1.10); *p* = 0.814	1.00 (0.92–1.09); *p* = 0.987	42.7 (40.0–45.4)	0.0 (−3.7 to 3.8); *p* = 0.987
36–47 months	1.13 (1.02–1.25); *p* = 0.024	1.12 (1.01–1.24); *p* = 0.025	47.9 (44.2–51.6)	5.2 (0.6 to 9.8); *p* = 0.027
48–59 months	1.20 (1.08–1.33); *p* < 0.001	1.20 (1.08–1.33); *p* < 0.001	51.1 (47.1–55.2)	8.4 (3.6 to 13.3); *p* < 0.001
**Child sex**				
Male	1.00 (ref.)	1.00 (ref.)	42.3 (40.5–44.1)	0.0 (ref.)
Female	1.04 (0.98–1.10); *p* = 0.166	1.05 (0.99–1.12); *p* = 0.076	44.6 (42.8–46.4)	2.3 (−0.2 to 4.8); *p* = 0.076
**Maternal age**				
15–19 years	1.04 (0.97–1.12); *p* = 0.293	1.01 (0.93–1.09); *p* = 0.895	43.4 (40.4–46.4)	0.2 (−3.2 to 3.7); *p* = 0.895
20–34 years	1.00 (ref.)	1.00 (ref.)	43.1 (41.5–44.8)	0.0 (ref.)
35–49 years	1.02 (0.94–1.10); *p* = 0.605	1.02 (0.94–1.11); *p* = 0.584	44.1 (41.1–47.1)	1.0 (−2.6 to 4.5); *p* = 0.586
**Maternal education**				
None	0.85 (0.61–1.21); *p* = 0.370	0.88 (0.62–1.26); *p* = 0.486	35.8 (23.3–48.3)	−4.8 (−17.7 to 8.0); *p* = 0.460
Primary	1.11 (1.02–1.21); *p* = 0.018	1.09 (0.99–1.21); *p* = 0.089	44.3 (41.3–47.4)	3.7 (−0.6 to 8.0); *p* = 0.090
Secondary	1.13 (1.05–1.22); *p* < 0.001	1.10 (1.02–1.19); *p* = 0.018	44.8 (43.0–46.5)	4.1 (0.8 to 7.5); *p* = 0.016
Higher	1.00 (ref.)	1.00 (ref.)	40.6 (37.9–43.3)	0.0 (ref.)
**Parity**				
1 child	1.00 (ref.)	1.00 (ref.)	42.5 (40.1–45.0)	0.0 (ref.)
2–3 children	1.02 (0.95–1.09); *p* = 0.658	1.02 (0.95–1.10); *p* = 0.568	43.4 (41.7–45.2)	0.9 (−2.2 to 4.0); *p* = 0.566
≥4 children	1.05 (0.97–1.15); *p* = 0.220	1.04 (0.95–1.14); *p* = 0.446	44.1 (41.1–47.0)	1.5 (−2.4 to 5.5); *p* = 0.447
**Wealth quintile**				
Poorest	1.14 (1.00–1.30); *p* = 0.044	1.20 (1.03–1.41); *p* = 0.020	46.0 (42.9–49.1)	7.8 (1.5 to 14.1); *p* = 0.015
Poorer	1.09 (0.96–1.25); *p* = 0.188	1.13 (0.98–1.31); *p* = 0.086	43.3 (40.9–45.6)	5.1 (−0.5 to 10.7); *p* = 0.074
Middle	1.11 (0.97–1.28); *p* = 0.130	1.15 (0.99–1.33); *p* = 0.065	43.8 (40.7–46.8)	5.6 (−0.1 to 11.4); *p* = 0.056
Richer	1.10 (0.95–1.28); *p* = 0.214	1.11 (0.95–1.30); *p* = 0.182	42.4 (38.5–46.3)	4.3 (−1.9 to 10.4); *p* = 0.175
Richest	1.00 (ref.)	1.00 (ref.)	38.2 (33.3–43.0)	0.0 (ref.)
**Area of residence**				
Urban	1.00 (ref.)	1.00 (ref.)	43.2 (41.7–44.7)	0.0 (ref.)
Rural	1.03 (0.98–1.09); *p* = 0.233	1.02 (0.94–1.09); *p* = 0.667	43.9 (41.2–46.6)	0.7 (−2.5 to 3.9); *p* = 0.668
**Natural region**				
Metropolitan Lima	1.00 (ref.)	1.00 (ref.)	47.4 (43.6–51.3)	0.0 (ref.)
Coast	0.89 (0.81–0.99); *p* = 0.024	0.86 (0.78–0.95); *p* = 0.003	41.0 (38.6–43.3)	−6.5 (−10.9 to −2.0); *p* = 0.004
Highlands	0.96 (0.88–1.05); *p* = 0.392	0.93 (0.84–1.03); *p* = 0.152	44.1 (42.0–46.3)	−3.3 (−7.8 to 1.3); *p* = 0.158
Jungle	0.90 (0.82–0.99); *p* = 0.033	0.86 (0.78–0.95); *p* = 0.004	40.9 (38.8–43.0)	−6.5 (−11.1 to −2.0); *p* = 0.005
**Health insurance**				
No	1.08 (0.99–1.18); *p* = 0.094	1.06 (0.96–1.16); *p* = 0.274	45.5 (41.4–49.6)	2.4 (−2.0 to 6.7); *p* = 0.284
Yes	1.00 (ref.)	1.00 (ref.)	43.2 (41.8–44.5)	0.0 (ref.)
**Survey year**				
2019	1.00 (ref.)	1.00 (ref.)	45.7 (43.0–48.4)	0.0 (ref.)
2020	1.03 (0.93–1.14); *p* = 0.569	1.00 (0.90–1.11); *p* = 0.994	45.7 (41.6–49.9)	0.0 (−4.9 to 4.9); *p* = 0.994
2021	0.89 (0.81–0.98); *p* = 0.019	0.91 (0.83–1.00); *p* = 0.048	41.6 (38.4–44.7)	−4.1 (−8.2 to −0.1); *p* = 0.045
2022	0.90 (0.82–0.99); *p* = 0.026	0.91 (0.83–0.99); *p* = 0.038	41.6 (38.7–44.4)	−4.1 (−8.0 to −0.3); *p* = 0.037
2023	0.93 (0.85–1.02); *p* = 0.119	0.93 (0.85–1.02); *p* = 0.111	42.5 (39.6–45.4)	−3.2 (−7.1 to 0.7); *p* = 0.110
2024	0.99 (0.91–1.09); *p* = 0.874	0.99 (0.91–1.09); *p* = 0.909	45.5 (42.4–48.5)	−0.2 (−4.3 to 3.8); *p* = 0.909
**Rainy season**				
No	1.00 (ref.)	1.00 (ref.)	42.8 (41.2–44.3)	0.0 (ref.)
Yes	1.07 (1.00–1.14); *p* = 0.041	1.05 (0.98–1.12); *p* = 0.168	44.7 (42.4–47.1)	2.0 (−0.9 to 4.8); *p* = 0.172

PR: prevalence ratio; pp: percentage points. The adjusted model included all variables shown (complete-case n = 11,493). Adjusted marginal prevalences and differences were obtained by marginal standardization; confidence intervals used the delta method with the full design-based covariance matrix.

## Data Availability

The microdata used are publicly available through the National Institute of Statistics and Informatics (INEI) repository: https://proyectos.inei.gob.pe/microdatos/(accessed on 15 July 2026).

## Supplementary Materials

The following supporting information can be downloaded at: https://www.mdpi.com/article/10.3390/children13101300/s1, Table S1: STROBE checklist for the cross-sectional study; Table S2: Operational definitions, original ENDES variables, denominators, and analytic use; Table S3: Overall and year-specific weighted prevalences of care seeking, treatment, and episode status; Table S4: Missing-data and treatment-denominator audit; Table S5: Alternative care-seeking indicators and care-cascade stages; Table S6: Sensitivity analyses for nutrition, wealth, zinc, and antibiotic definitions; Table S7: Reviewer-requested verification analyses; Figure S1: Flow diagram for analytical sample selection.
