# Supplementary material for: Gaps in Healthcare Seeking for Childhood Diarrhea Among Peruvian Children Under Five Years of Age: Socioeconomic and Clinical Patterns in a National Survey
Source: Children (Basel). 2026 Sep 25;13(10):1300. doi: 10.3390/children13101300 (PMC13703355; doi:10.3390/children13101300)
Supplement: Supplementary file 1 [file children-13-01300-s001.zip › children-4330275-supplementary.pdf]

Supplementary Materials

Supplementary Table S1. STROBE checklist for the cross-sectional study.

No.	Recommendation	Location in the manuscript
TITLE AND ABSTRACT	TITLE AND ABSTRACT	TITLE AND ABSTRACT
1(a)	Indicate the study design in the title or abstract.	Title identifies a national survey; Abstract and Methods identify an analytical cross-sectional analysis of repeated ENDES rounds.
1(b)	Provide an informative and balanced abstract.	Single-paragraph abstract reports the objective, full survey-design methods, n=11,942, non-use 43.5%, restrictive formal care 33.1%, non-formal-only care 23.4%, ORS 34.3%, zinc 1.0%, ORS+zinc 0.5%, and the principal adjusted associations.
INTRODUCTION	INTRODUCTION	INTRODUCTION
2	Explain scientific background and rationale.	Introduction describes global and regional burden, recommended ORS/zinc management, persistent care and treatment gaps, and the Peruvian evidence gap in the post-episode care pathway.
3	State specific objectives and prespecified hypotheses.	Introduction final paragraph states the primary objective (non-use of external care and associated factors) and secondary objectives (formal/non-formal sources, treatment indicators, and diarrhea still active).
METHODS	METHODS	METHODS
4	Present key elements of study design early.	Section 2.1: analytical cross-sectional study using ENDES 2019–2024.
5	Describe setting, locations, dates, and data collection.	Section 2.2: continuous national ENDES survey, Peru, six annual rounds, probabilistic stratified multistage sampling, standardized household interviews.
6(a)	State participant eligibility and selection methods.	Section 2.3 and Figure S1: children aged 0–59 months, recent diarrhea, classifiable H12 care-seeking status; complete cases for adjusted models.
7	Define outcomes, exposures, predictors, confounders, and effect modifiers.	Section 2.4 and Table S2 provide original ENDES variables, coding, denominators, and analytic roles for H12, H44B/H44C, ORS, zinc, antibiotics, warning features, covariates, stunting, and wasting.
8	Describe data sources and measurement.	Sections 2.2–2.4: standardized ENDES child/household modules and original variable codes; H44C wording and H46B exclusion are explicit.
9	Describe efforts to address bias.	Full survey-design estimation, domain analysis, prespecified covariates, complete-case reporting, source-variable audits, year-specific treatment estimates, sequential secondary models, and cautious non-causal interpretation.
10	Explain study size.	Section 3.1 and Figure S1: 100,119 identified; 12,180 with recent diarrhea; 11,942 classifiable main sample; 11,493 main model complete cases; 11,921 primary secondary-outcome model.
11	Explain handling of quantitative variables and categories.	Section 2.4: age, maternal age, parity, wealth, and other covariates use prespecified ENDES-compatible categories.
12(a)	Describe statistical methods and control of confounding.	Section 2.5: StataNow/SE 19.5 svy commands, Taylor linearization, modified Poisson regression, a priori adjustment set, marginal standardization, and delta-method confidence intervals using the full design covariance.
12(b)	Describe subgroup and interaction methods.	Ordinal wealth trend and warning-feature-by-wealth interaction; Figure 2 estimates and contrasts in Tables S6–S7.
12(c)	Explain handling of missing data.	Complete-case regression; denominators and missingness shown in Tables S3–S4.
12(d)	Explain how sampling design was addressed.	Annual weights normalized by six; year-specific PSU/strata; singleton strata centered; no FPC; domain analysis; design effect and survey diagnostics in Table S7.
12(e)	Describe sensitivity analyses.	Nutrition adjustment, zinc/antibiotic source definitions, expanded M4 model, wealth trend/interaction, and source-data verification in Tables S6–S7. LCA was removed.
RESULTS	RESULTS	RESULTS
13(a)	Report numbers at each study stage.	Section 3.1 and Figure S1 report all selection stages.
13(b)	Give reasons for exclusions.	The 238 exclusions had no affirmative external source and missing/non-informative H12Y; no internal H12 contradiction was found.
13(c)	Consider a flow diagram.	Supplementary Figure S1.
14(a)	Present descriptive characteristics.	Table 1 presents unweighted n and weighted column percentages.
14(b)	Report missing data for each variable.	Tables S3–S4 report variable-specific denominators and missingness.
15	Report outcome events or summary measures.	Section 3.3 and Table S3 report non-use 43.5%, formal care 33.1%, non-formal-only 23.4%, ORS 34.3%, zinc 1.0%, ORS+zinc 0.5%, antibiotics 17.7%, same/next-day care 69.5%, and active diarrhea 14.6%.
16(a)	Report unadjusted and adjusted estimates with precision and confounders.	Table 2 reports crude PRs, aPRs, p values, adjusted marginal prevalences, and adjusted differences; the full adjustment set is stated in Section 2.5.
16(b)	Report category boundaries.	Categories are shown in Tables 1–2 and Table S2.
16(c)	Translate relative measures into absolute measures when relevant.	Table 2 reports adjusted prevalence differences in percentage points.
17	Report other analyses.	Figure 2, sequential M0–M4 secondary models, nutrition sensitivity, H12/H44B/H44C audits, design diagnostics, and source-variable analyses are reported in Tables S6–S7.
DISCUSSION	DISCUSSION	DISCUSSION
18	Summarize key results in relation to objectives.	Section 4.1 summarizes care-cascade, non-formal source, treatment, clinical, age, and wealth findings.
19	Discuss limitations and potential bias.	Section 4.4 addresses cross-sectional temporality, reverse causality, self-report, H44C interpretation, variable-specific treatment denominators, source-label discrepancies, harmonization, and complete-case assumptions.
20	Give cautious overall interpretation.	Discussion avoids causal language, avoids claiming a monotonic socioeconomic gradient or effect modification, and separates descriptive, adjusted, and sensitivity results.
21	Discuss generalizability.	Section 4.3 discusses relevance to middle-income health systems with declining mortality but incomplete effective coverage.
OTHER INFORMATION	OTHER INFORMATION	OTHER INFORMATION
22	Report funding and funder role.	Funding declaration states that APCs may be covered by UNTRM and that the funder had no role in design, collection, analysis, interpretation, or manuscript preparation; data are publicly available through INEI.

STROBE = Strengthening the Reporting of Observational Studies in Epidemiology.

Supplementary Table S2. Operational definitions, original ENDES variables, denominators, and analytic use.

Construct	Clean/derived variable	Original ENDES variable(s)	Operational definition/coding rule	Denominator	Analytic use
Recent diarrhea	muestra_diarrea	H11	Caregiver-reported diarrhea in the preceding two weeks. Original annual REC43 audit found no H11=1 observations; all affirmative records were H11=2.	12,180	Eligibility
Classifiable care seeking	muestra_principal	H12A–H12X, H12Y, H12Z	Recent diarrhea plus classifiable external-care status.	11,942	Main analytic sample
Any external care seeking	busco_atencion_externa	H12A–H12X/H12Z	At least one source-specific affirmative response.	11,942	Complement of primary outcome
Non-use of external care	no_busco_atencion	H12A–H12X/H12Z; H12Y	No source-specific affirmative response and H12Y indicating no advice/care/treatment. Source-specific affirmative response would take precedence in a conflict; none occurred.	11,942	Primary outcome
Restrictive formal care	busco_formal_restrictiva	H12A–H12J, H12L–H12N, H12P–H12Q, H12V, H12W, H12Z	External source excluding pharmacy/botica, direct purchase, friend/relative, traditional, and other non-medical sources.	11,942	Secondary care-source outcome
Broad formal care	busco_formal_amplia	H12A–H12Q, H12V, H12W, H12Z	Health-sector source using a broader source list.	11,942	Alternative definition
Non-formal-only external care	solo_no_formal	H12 block + restrictive formal definition	Any external source without a restrictive formal source.	11,942	Care-cascade intermediate group
Pharmacy/direct purchase	busco_farmacia_compra	H12K, H12O, H12S	Pharmacy, botica/botiquín, or direct-purchase source; H12S was structurally nonpopulated.	12,180	Source indicator
Blood in stool	sangre_heces	H11B	Caregiver-reported blood in stool.	11,933	Clinical-need marker
Co-reported fever	fiebre_coreportada	H22/H22-derived fever item	Fever reported in the same reference period; not clinically attributed to diarrhea.	11,939	Clinical-need marker
Survey-defined warning features	signo_alarma	H11B + fever item	Blood in stool and/or co-reported fever. Not a complete clinical danger-sign assessment.	11,942	Descriptive stratifier/interaction
Diarrhea still active	diarrea_activa	H44C	Questionnaire wording: whether the child "still has diarrhea" at interview.	11,933	Secondary outcome; not duration
Time to care	h44b_valido; atencion_0_1_dias	H44B	Days from diarrhea onset to seeking advice/treatment; same/next day = 0 or 1 day.	6,690 care seekers	Timeliness indicator
Fever timing variable excluded	—	H46B	Interval before seeking advice/treatment for fever; not continuity of diarrhea.	Not used	Clarification
Oral rehydration salts	recibio_sro	H13 and/or H13B	ORS packet or prepackaged ORS liquid reported for the episode.	11,932	Treatment outcome
Zinc: primary	recibio_zinc	S473DA and S473DB	Specific zinc-tablet or zinc-syrup items populated in all six rounds.	12,147	Primary zinc outcome
Zinc: sensitivity	zinc_h15e	H15E	Official-dictionary zinc mapping examined only as sensitivity because the integrated-file label differed.	12,180	Source-variable sensitivity
ORS + zinc	recibio_sro_zinc	ORS variables + S473DA/S473DB	Both ORS and primary zinc indicators affirmative.	11,899	Treatment-quality outcome
Antibiotics: primary	recibio_antibiotico	H15 and H15B	Antibiotics according to official annual dictionaries; H15B was structurally empty.	12,174	Primary antibiotic indicator
Diarrhea syrup	recibio_jarabe	H15A	Jarabe para la diarrea; not classified as antibiotic or zinc.	12,174	Sensitivity/source audit
Home remedy	recibio_remedio_casero	H15D	Caregiver-reported home remedy.	12,174	Treatment outcome
Child age	grupo_edad	B8/age in months	0–11, 12–23, 24–35, 36–47, 48–59 months.	11,942	Covariate
Child sex	sexo_nino	B4	Male/female.	11,942	Covariate
Maternal age	edad_madre_grupo	V012	15–19, 20–34, 35–49 years.	11,865	Covariate
Maternal education	educacion_madre	V106	None, primary, secondary, higher.	11,865	Covariate
Parity	paridad_cat	V201	1, 2–3, ≥4 children.	11,580	Covariate
Wealth quintile	riqueza	V190	Poorest, poorer, middle, richer, richest.	11,942	Covariate/trend
Area of residence	area_rural	V025	Urban/rural.	11,942	Covariate
Natural region	region_natural	SREGION/SHREGION	Metropolitan Lima, Coast, Highlands, Jungle.	11,942	Covariate
Health insurance	tiene_seguro	V481	Any reported health insurance.	11,942	Covariate
Rainy season	estacion_lluviosa	Interview month	Prespecified month-based indicator.	11,942	Covariate
Stunting	stunting	HW70	Height-for-age z score < −2.	11,358 in sensitivity model	Background vulnerability sensitivity
Wasting	wasting	HW72	Weight-for-height z score < −2.	11,358 in sensitivity model	Background vulnerability sensitivity
Pooled weight	peso_pooled	Annual survey weight	Annual weight divided by six only to normalize pooled scale; sum not interpreted as population count.	All records	Survey estimation
Pooled PSU/stratum	psu_pool; strata_pool	Annual PSU/stratum + year	Unique year-specific design identifiers; Taylor linearization; singleton strata centered; no FPC.	Full design/domain	Survey variance

Note. Variable labels and coding rules were audited against the original annual REC43 files and official INEI dictionaries. Treatment-specific denominators differ from the H12-classifiable main sample.

Supplementary Table S3. Overall and year-specific weighted prevalences of care seeking, treatment, and episode status.

S3A. Overall indicators.

Indicator	Source/clean variable	Unweighted denominator	Unweighted events	Weighted prevalence, % (95% CI)
Non-use of external care	H12-derived	11,942	5,252	43.5 (42.2–44.8)
Any external care	H12-derived	11,942	6,690	56.5 (55.2–57.8)
Restrictive formal care	H12 restrictive	11,942	3,899	33.1 (31.8–34.3)
Non-formal-only external care	H12-derived	11,942	2,791	23.4 (22.3–24.5)
ORS	H13/H13B	11,932	3,976	34.3 (33.1–35.6)
Zinc-specific	S473DA/S473DB	12,147	120	1.0 (0.8–1.2)
ORS + zinc	Combined	11,899	59	0.5 (0.3–0.7)
Antibiotics	H15/H15B	12,174	2,276	17.7 (16.7–18.6)
Home remedies	H15D	12,174	3,108	22.3 (21.3–23.3)
Pharmacy/direct purchase	H12K/H12O/H12S	12,180	2,278	18.6 (17.6–19.6)
Same-day or next-day care	H44B among care seekers	6,690	4,606	69.5 (67.9–71.0)
Diarrhea still active	H44C	11,933	1,825	14.6 (13.7–15.6)

Treatment indicators use variable-specific valid denominators among the 12,180 recent-diarrhea observations; care-seeking indicators use the 11,942 H12-classifiable observations.

S3B. Year-specific ORS, zinc, and joint ORS + zinc receipt.

Indicator	Survey year	Valid denominator	Weighted prevalence, % (95% CI)
ORS	2019	2,203	33.3 (30.8–35.9)
ORS	2020	940	31.0 (27.1–34.8)
ORS	2021	1,886	34.7 (31.6–37.9)
ORS	2022	2,331	32.7 (29.9–35.4)
ORS	2023	2,419	36.1 (33.2–39.0)
ORS	2024	2,153	35.4 (32.5–38.4)
ORS	Pooled	11,932	34.3 (33.1–35.6)
Zinc-specific	2019	2,197	0.4 (0.0–0.7)
Zinc-specific	2020	1,082	1.2 (0.3–2.1)
Zinc-specific	2021	1,979	1.2 (0.4–2.0)
Zinc-specific	2022	2,331	1.2 (0.7–1.7)
Zinc-specific	2023	2,408	1.2 (0.6–1.8)
Zinc-specific	2024	2,150	0.7 (0.4–1.1)
Zinc-specific	Pooled	12,147	1.0 (0.8–1.2)
ORS plus zinc	2019	2,194	0.2 (0.0–0.4)
ORS plus zinc	2020	939	0.8 (0.0–1.7)
ORS plus zinc	2021	1,882	0.7 (0.1–1.3)
ORS plus zinc	2022	2,328	0.4 (0.1–0.7)
ORS plus zinc	2023	2,407	0.7 (0.2–1.1)
ORS plus zinc	2024	2,149	0.3 (0.1–0.6)
ORS plus zinc	Pooled	11,899	0.5 (0.3–0.7)

The specific zinc variables S473DA/S473DB were present and had affirmative observations in every annual source file. The lower confidence bound is displayed as 0.0% when the linearized estimate rounds below 0.05%.

Supplementary Table S4. Missing-data and treatment-denominator audit.

S4A. Main H12-classifiable sample (n=11,942).

Variable/model	Missing, n	Missing, %	Valid observations
External care seeking	0	0.0	11,942
Blood in stool	9	0.1	11,933
Co-reported fever	3	<0.1	11,939
Survey-defined warning features	0	0.0	11,942
Child age	0	0.0	11,942
Child sex	0	0.0	11,942
Maternal age	77	0.6	11,865
Maternal education	77	0.6	11,865
Parity	362	3.0	11,580
Wealth quintile	0	0.0	11,942
Area of residence	0	0.0	11,942
Natural region	0	0.0	11,942
Health insurance	0	0.0	11,942
Survey year	0	0.0	11,942
Rainy season	0	0.0	11,942
Diarrhea still active (H44C)	9	0.1	11,933
Complete main adjusted model	449	3.8	11,493
Complete primary secondary model M3	21	0.2	11,921

S4B. Treatment-specific denominators among 12,180 recent-diarrhea observations.

Indicator	Missing/non-valid, n	Valid denominator
ORS	248	11,932
Zinc-specific	33	12,147
ORS + zinc	281	11,899
Antibiotics	6	12,174
Home remedies	6	12,174
Pharmacy/direct purchase	0	12,180
H44B among 6,690 external-care seekers	0	6,690

The 238 H12-unclassifiable observations were excluded from the primary care-seeking analysis because no external source was affirmative and H12Y was missing/non-informative; none was internally contradictory.

Supplementary Table S5. Alternative care-seeking indicators and care-cascade stages.

Definition/indicator	Unweighted denominator	Weighted prevalence, % (95% CI)	Interpretation
Non-use of any external care	11,942	43.5 (42.2–44.8)	Primary outcome
Any external care	11,942	56.5 (55.2–57.8)	Complement of primary outcome
Restrictive formal care seeking	11,942	33.1 (31.8–34.3)	Formal facility/provider definition
Non-use of restrictive formal care	11,942	66.9 (65.7–68.2)	Complement of restrictive formal care
Only non-formal external sources	11,942	23.4 (22.3–24.5)	External contact without restrictive formal source
Only non-formal sources among any external-care seekers	6,690	41.4 (39.7–43.1)	Conditional care-cascade measure
Pharmacy/direct-purchase source	12,180	18.6 (17.6–19.6)	Source-specific indicator

Supplementary Table S6. Sensitivity analyses for nutrition, wealth, zinc, and antibiotic definitions.

S6A. Nutrition sensitivity of selected main-model associations.

Term	Main model, n=11,493	Anthropometry-complete baseline, n=11,358	Additionally adjusted for stunting	Additionally adjusted for wasting
Blood in stool: yes vs no	0.52 (0.42–0.63)	0.52 (0.42–0.63)	0.52 (0.42–0.63)	0.52 (0.42–0.63)
Co-reported fever: yes vs no	0.80 (0.75–0.85)	0.79 (0.75–0.85)	0.79 (0.75–0.85)	0.80 (0.75–0.85)
Poorest vs richest quintile	1.20 (1.03–1.41)	1.22 (1.04–1.43)	1.22 (1.04–1.43)	1.22 (1.04–1.43)

Independent nutritional-status associations.

Nutritional indicator	aPR (95% CI)	p-value	n
Stunting: yes vs no	0.99 (0.91–1.07)	0.803	11,358
Wasting: yes vs no	0.71 (0.46–1.10)	0.125	11,358

S6B. Wealth trend and warning-feature interaction.

Analysis/stratum	Contrast	Estimate	95% CI	p-value
Ordinal wealth trend	Per one-level shift toward greater poverty	aPR 1.04	1.00–1.07	0.041
Deviation from linearity	Joint test of categorical departures	—	—	0.583
Warning-feature × wealth interaction	Global interaction test	—	—	0.186
No warning features	Poorest vs richest	aPR 1.11; adjusted difference 5.2 pp	aPR 0.94–1.33; difference −2.9 to 13.3	0.225
Warning features	Poorest vs richest	aPR 1.38; adjusted difference 11.3 pp	aPR 1.06–1.80; difference 3.0–19.5	0.017

S6C. Zinc and antibiotic source-variable definitions.

Definition	Original variable(s)	Source of definition	n	Weighted prevalence, %	95% CI	Interpretation
Main zinc definition	S473DA or S473DB	Populated specific zinc items in integrated ENDES file	12147	1.0	0.8–1.2	Primary estimate; zinc tablets or zinc syrup.
Official-dictionary H15E sensitivity	H15E	Official INEI 2024 dictionary	12180	1.1	0.9–1.4	Sensitivity only because the integrated file labels H15E differently.
Broad zinc sensitivity	S473DA or S473DB or H15E	Union of specific zinc items and official-dictionary H15E	12147	1.1	0.9–1.4	Upper-bound sensitivity across discordant mappings.
Main INEI-dictionary definition	H15 or H15B	Official INEI 2024 dictionary	12174	17.7	16.7–18.6	Primary estimate; H15B is not populated in the integrated file, so the estimate is driven by H15.
Integrated-file label definition	H15A	Variable label in supplied integrated DTA	12174	26.9	25.8–28.0	Sensitivity only; the official 2024 dictionary identifies H15A as diarrhea syrup, not antibiotic.
Broad sensitivity definition	H15 or H15A or H15B	Union of official-dictionary and integrated-label mappings	12174	41.8	40.5–43.1	Upper-bound sensitivity combining discordant source mappings.

The main manuscript uses S473DA/S473DB for zinc and H15/H15B for antibiotics. H15A is diarrhea syrup and is not included in the primary antibiotic definition.

Supplementary Table S7. Reviewer-requested verification analyses.

S7A. Figure 2 point estimates and design-based 95% confidence intervals.

Warning-feature stratum	Wealth quintile	n	Weighted prevalence, %	95% CI	PSUs	Strata
No warning features	Poorest	2,048	49.7	46.9–52.4	9019	423
No warning features	Poorer	1,980	48.0	44.9–51.1	13249	776
No warning features	Middle	1,284	51.0	47.2–54.8	11167	666
No warning features	Richer	824	49.4	44.3–54.6	7661	472
No warning features	Richest	491	46.1	40.0–52.3	5360	311
Warning features	Poorest	1,847	40.2	37.5–42.9	8075	375
Warning features	Poorer	1,596	37.3	33.8–40.7	12663	693
Warning features	Middle	962	34.7	30.0–39.4	9871	578
Warning features	Richer	583	35.5	29.9–41.1	6527	378
Warning features	Richest	327	30.3	23.2–37.5	3997	212

S7B. H12 source-by-H12Y consistency audit.

Any source status	H12Y status/summary	n	Weighted % of all recent-diarrhea records
Any external source=0	H12Y no treatment=0	0	0.0
Any external source=0	H12Y no treatment=1	5,252	42.8
Any external source=1	H12Y no treatment=0	6,690	55.5
Any external source=1	H12Y no treatment=1	0	0.0
Summary	Contradictory records	0	
Summary	H12-classifiable	11,942	
Summary	H12-unclassifiable	238	

No source-specific affirmative response co-occurred with H12Y indicating no treatment. The 238 unclassifiable records lacked an affirmative source and had missing/non-informative H12Y.

S7C. Original-file H11 audit and H11-by-H44C cross-check.

Year	H11=0, n (weighted %)	H11=1, n (weighted %)	H11=2, n (weighted %)	H11=8, n (weighted %)	Missing, n (weighted %)
2019	18,405 (86.9)	0 (0.0)	2,483 (11.3)	49 (0.4)	217 (1.4)
2020	15,598 (90.3)	0 (0.0)	1,454 (8.1)	22 (0.1)	168 (1.6)
2021	19,633 (88.7)	0 (0.0)	2,233 (9.2)	33 (0.2)	201 (1.9)
2022	18,750 (86.2)	0 (0.0)	2,619 (11.5)	31 (0.2)	211 (2.1)
2023	17,940 (85.4)	0 (0.0)	2,689 (12.6)	23 (0.2)	188 (1.8)
2024	17,170 (86.6)	0 (0.0)	2,394 (11.7)	18 (0.3)	169 (1.4)

H11 category	H44C no	H44C yes	H44C missing	Total	% H44C yes	95% CI
H11=0 no recent diarrhea	0	0	87697	87697	NA	NA–NA
H11=1 last 24 hours	0	0	0	0	NA	NA–NA
H11=2 last two weeks	10108	1825	247	12180	0.146	0.137–0.156
H11=8 don't know	0	0	33	33	NA	NA–NA
H11 missing	0	0	209	209	NA	NA–NA

All affirmative recent-diarrhea records in the original annual files were H11=2; no H11=1 record was present. H44C was therefore retained only as current episode status, not total duration.

S7D. Sequential models for diarrhea still active.

Model	Role	Adjustment set	PR	95% CI	p-value	n
M0	Sequential	Non-use of external care only	0.95	0.84–1.08	0.464	11,933
M1	Sequential	M0 plus blood in stool	0.96	0.85–1.09	0.509	11,924
M2	Sequential	M0 plus co-reported fever	0.99	0.87–1.12	0.826	11,930
M3	Primary	M0 plus blood in stool and co-reported fever	0.99	0.87–1.12	0.848	11,921
M4	Sensitivity	M3 plus child, maternal, socioeconomic, geographic, year, and season covariates	1.00	0.88–1.13	0.995	11,485

Supplementary Table S7. Reviewer-requested verification analyses (continued).

S7E. H44B timing-item audit.

Metric	Value
External-care seekers	6690.000
Valid H44B	6690.000
Missing	0.000
Don't know	0.000
Not applicable	0.000
Day 0	1852.000
Day 1	2754.000
Day 2 or later	2084.000
Same/next day prevalence	0.695
Standard error	0.008
95% CI lower	0.679
95% CI upper	0.710

S7F. Non-formal-only source prevalence and composition.

Indicator	Category/denominator	n	Weighted prevalence, %	95% CI
Nonformal-only	All recent diarrhea	11,942	23.4	22.3–24.5
Nonformal-only	Among external care seekers	6,690	41.4	39.7–43.1
nf_farmacia_botica	Composition within nonformal-only	2,791	76.4	73.7–79.1
nf_compra_directa	Source variable not populated	0	NA	NA
nf_amigo_pariente	Composition within nonformal-only	2,791	22.8	20.1–25.4
nf_tradicional	Composition within nonformal-only	2,791	0.8	0.4–1.2
nf_otra_fuente	Composition within nonformal-only	2,791	2.1	1.2–3.0
nf_multiples	Composition within nonformal-only	2,791	2.0	1.3–2.8

Component	Original variable(s)	Source/definition	n	Weighted prevalence, %	95% CI	Interpretation
Main INEI-dictionary definition	H15 or H15B	Official INEI 2024 dictionary	12174	17.7	16.7–18.6	Primary estimate; H15B is not populated in the integrated file, so the estimate is driven by H15.
Integrated-file label definition	H15A	Variable label in supplied integrated DTA	12174	26.9	25.8–28.0	Sensitivity only; the official 2024 dictionary identifies H15A as diarrhea syrup, not antibiotic.
Broad sensitivity definition	H15 or H15A or H15B	Union of official-dictionary and integrated-label mappings	12174	41.8	40.5–43.1	Upper-bound sensitivity combining discordant source mappings.

S7G. Survey-design diagnostics for the primary prevalence.

Metric	Value
Unweighted n	11942.000
Sum of normalized pooled weights	1109.149
Prevalence	0.435
Standard error	0.007
95% CI lower	0.422
95% CI upper	0.448
PSUs retained by total design	17978.000
PSUs with subpopulation observations	8287.000
Strata	1311.000
Design degrees of freedom	16667.000
Singleton strata	38.000
Omitted strata	142.000
Design effect	2.065
DEFT	1.437

S7H. Category-specific row prevalences of non-use cited in the manuscript.

Variable	Category	n category	n non-use	Weighted non-use, %	95% CI
Blood in stool	No	11,231	5,080	44.8	43.5–46.1
Blood in stool	Yes	702	167	22.1	17.8–26.4
Co-reported fever	No	6,942	3,369	48.0	46.3–49.7
Co-reported fever	Yes	4,997	1,883	37.4	35.4–39.4
Survey-defined warning features	No	6,627	3,286	49.0	47.3–50.7
Survey-defined warning features	Yes	5,315	1,966	36.6	34.7–38.5
Household wealth quintile	Poorest	3,895	1,750	45.1	43.2–47.1
Household wealth quintile	Poorer	3,576	1,545	43.2	40.9–45.6
Household wealth quintile	Middle	2,246	1,008	44.0	41.0–47.1
Household wealth quintile	Richer	1,407	621	43.6	39.7–47.5
Household wealth quintile	Richest	818	328	39.6	34.8–44.4

Supplementary Table S7. Reviewer-requested verification analyses (continued).

S7I. Annual antibiotic variable audit.

Year	H15 nonmissing	H15 yes	H15A nonmissing	H15A yes	H15B nonmissing	H15B yes
2019	2483	558	2483	603	0	0
2020	1454	224	1454	266	0	0
2021	2233	396	2233	635	0	0
2022	2619	438	2619	740	0	0
2023	2689	466	2689	761	0	0
2024	2394	473	2394	679	0	0

Official annual labels consistently identified H15 as antibiotics, H15A as diarrhea syrup, and H15B as antibiotic injection; H15B was structurally empty in all six rounds.

S7J. Software versions and roles.

Software/package	Version	Role
StataNow/SE	19.5 (Revision 21 May 2025)	Primary estimation; svy Taylor linearization
Python	3.12.0	Output verification and orchestration
pandas	3.0.3	Organization of Stata-exported results
NumPy	2.4.6	Numeric handling; not survey estimation
patsy	1.0.2	Not used in final estimation
statsmodels	0.14.6	Not used in final estimation
matplotlib	3.11.0	Figure rendering from Stata estimates

Supplementary Figure S1. Flow diagram for analytical sample selection.

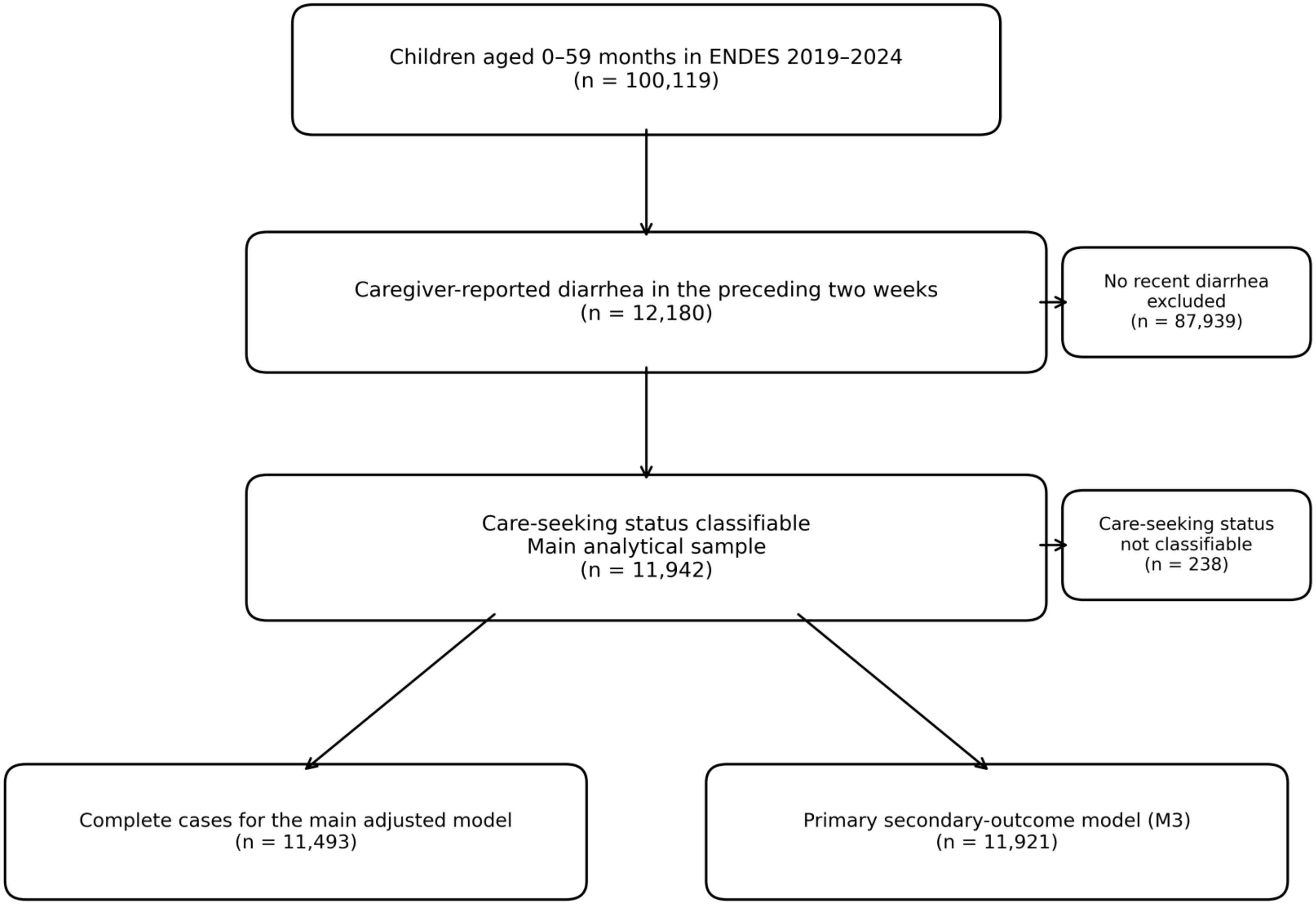

Supplementary Figure S1. Flow diagram for analytical sample selection.

The main analytical sample required classifiable H12 care-seeking information. M3 was the prespecified secondary-outcome model adjusted for blood in stool and co-reported fever.

Abbreviations: aPR, adjusted prevalence ratio; CI, confidence interval; ENDES, Demographic and Family Health Survey; FPC, finite population correction; ORS, oral rehydration salts; PR, prevalence ratio; PSU, primary sampling unit.
